# Self-reported arm and shoulder problems in breast cancer survivors in Sub-Saharan Africa: the African Breast Cancer-Disparities in Outcomes cohort study

**DOI:** 10.1186/s13058-021-01486-9

**Published:** 2021-11-24

**Authors:** Pauline Boucheron, Angelica Anele, Annelle Zietsman, Moses Galukande, Groesbeck Parham, Leeya F. Pinder, Therese M.-L. Andersson, Benjamin O. Anderson, Milena Foerster, Joachim Schüz, Isabel dos Santos Silva, Valerie McCormack

**Affiliations:** 1 International Agency for Research On Cancer (IARC/WHO), Environment and Lifestyle Epidemiology Branch, Lyon, France; 2 FMC, Owerri, Nigeria; 3 AB May Cancer Centre, Windhoek Central Hospital, Windhoek, Namibia; 4 College of Health Sciences, Makerere University, Kampala, Uganda; 5 Department of Obstetrics and Gynaecology, School of Medicine, University of North Carolina, Chapel Hill, NC USA; 6 University of Washington, Seattle, WA USA; 7 Department of Medical Epidemiology and Biostatistics, Karolinska Institutet, Stockholm, Sweden; 8 World Health Organization, Geneva, Switzerland; 9 London School of Hygiene and Tropical Medicine (LSHTM), London, UK

**Keywords:** Arm and shoulder problems, Lymphedema, Lymphoedema, Lymphodema, Sub-Saharan Africa, Breast cancer, Low- and middle-income countries, Arm swelling, Arm stiffness, Shoulder pain

## Abstract

**Background:**

Arm and shoulder problems (ASP), including lymphedema, were common among women with breast cancer in high-income countries before sentinel lymph node biopsy became the standard of care. Although ASP impair quality of life, as they affect daily life activities, their frequency and determinants in Sub-Saharan Africa remain unclear.

**Methods:**

All women newly diagnosed with breast cancer at the Namibian, Ugandan, Nigerian, and Zambian sites of the African Breast Cancer-Disparities in Outcomes (ABC-DO) cohort study were included. At each 3-month follow-up interview, women answered the EORTC-QLQ-Br23 questionnaire, including three ASP items: shoulder/arm pain, arm stiffness, and arm/hand swelling. We estimated the cumulative incidence of first self-reported ASP, overall and stratified by study and treatment status, with deaths treated as competing events. To identify determinants of ASP, we estimated cause-specific hazard ratios using Cox models stratified by study site.

**Results:**

Among 1476 women, up to 4 years after diagnosis, 43% (95% CI 40–46), 36% (33–38) and 23% (20–25), respectively, self-reported having experienced arm/shoulder pain, stiffness and arm/hand swelling at least once. Although risks of self-reported ASP differed between sites, a more advanced breast cancer stage at diagnosis, having a lower socioeconomic position and receiving treatment increased the risk of reporting an ASP.

**Conclusion:**

ASP are very common in breast cancer survivors in Sub-Saharan Africa. They are influenced by different factors than those observed in high-income countries. There is a need to raise awareness and improve management of ASP within the African setting.

**Supplementary Information:**

The online version contains supplementary material available at 10.1186/s13058-021-01486-9.

## Background

Breast cancer is the most commonly diagnosed cancer in women worldwide [1]. In 2020, 129,000 Sub-Saharan African women were newly diagnosed with this cancer, and the incidence is projected to increase [1]. Before the development of sentinel lymph node biopsy, in high-income countries (HICs), arm and shoulder problems (ASP), defined by stiffness, pain and swelling, were frequent among breast cancer patients and survivors. In this context, ASP were typically ipsilateral and occurred due to axillary lymph node dissection, mastectomy, chemotherapy, and radiotherapy. ASP risk factors also included number of lymph nodes removed, older age, and high body mass index (BMI) [2–4]. Although ASP are not life threatening, they impair women’s quality of life in the long-term as they affect daily life and activities [5].

In Sub-Saharan Africa (SSA), the frequency of ASP in breast cancer survivors is unclear. A recent meta-analysis that aimed to assess the prevalence and incidence of lymphedema (i.e. arm swelling) in low- and middle-income countries (LMICs), which included only one study from SSA, could not obtain a pooled estimate because of large between-study heterogeneity [6]. This was due to the differences in study designs, in measurement methods and definitions, and duration of follow-up. Since this meta-analysis, a few additional studies from SSA have been published but none was designed to estimate the frequency of ASP, and they were too small to allow a thorough investigation of ASP risk factors [4, 7–10].

Risk factors for ASP in SSA may differ from that of HICs because of the very advanced stage at breast cancer diagnosis, different treatment courses or the broader environment including the physical burden in daily life or infectious agents [11–13]. In this setting, a substantial proportion of women do not receive timely, complete or high-quality treatment [14]. For instance, surgical procedures commonly used in HICs, such as sentinel lymph node biopsy and breast-conserving surgery, are less often performed in SSA where mastectomy and axillary lymph node dissection are the predominant surgical procedures. Moreover, ASP can already be present at the time of diagnosis, before treatment initiation, due to the physical impact of extremely large tumour sizes and high number of affected lymph nodes at diagnosis. Furthermore, it is conceivable that risk factors may vary across different ethnic groups.

In this context, the present study aimed to estimate the frequency and determinants of ASP after a breast cancer diagnosis within the African Breast Cancer-Disparities in Outcomes (ABC-DO) cohort, a prospective cohort of women with breast cancer in five SSA countries.

## Methods

### Study design and data collection

This study was part of the African Breast Cancer-Disparities in Outcomes (ABC-DO) study, a prospective multicentric hospital-based cohort study of disparities in survival in women after a diagnosis of breast cancer. ABC-DO was conducted in Nigeria, Zambia, Uganda, Namibia and South Africa and its protocol is available elsewhere [15]. In brief, from September 2014 to early 2017, all women aged 18 and above who visited one of the participating hospitals and were suspected of having breast cancer were invited to participate. Of 2313 women recruited with suspected breast cancer, 2228 had the disease confirmed by histology, cytology or clinically. Overall, 2212 (99%) of those eligible women accepted to participate and were included in ABC-DO.

### Data collection

At baseline, data on women’s sociodemographic characteristics, comorbidities, breast cancer risk factors and health attitudes, knowledge and beliefs were collected via an interviewer-administered questionnaire. The woman agreed to be contacted thereafter every three months by mobile phone. MHealth technology was used for all real-time data collection and to facilitate contact with women or their next of kin every three-months, using a standardized protocol that minimized losses to follow-up [16]. At each 3-month follow-up contact, data on treatment (surgery, radiotherapy, chemotherapy, and endocrine therapy) were collected from both medical records and self-reports. The woman also answered the EORTC-QLQ-Br23 questionnaire, an internationally validated questionnaire to assess quality of life of women with breast cancer [17]. For the primary ASP outcomes, we used responses to the three questions pertaining, at each trimonthly contact, to the past week “Did you have pain in your arm or shoulder?”; “Did you have a swollen arm or hand?” and “Was it difficult to raise your arm or to move it sideways?” for information on arm or shoulder pain, arm or hand swelling and arm stiffness, respectively. Each of the three items were rated on a four-point Likert scale of “not at all”, “a little”, “quite a bit” and “very much”. In the present analysis, each ASP was considered present if rated “quite a bit” or “very much” and absent otherwise, as was previously done in other studies [5, 18, 19].

### Inclusion criteria and exclusion criteria

For the purpose of this analysis, all women enrolled into ABC-DO were included except those from South Africa (*n* = 675) because the regular follow-up at this site did not systematically ascertain ASP. Prevalent breast cancer cases (i.e. women with a previous diagnosis of breast cancer more than two years before enrolment *n* = 57) or who were lost to follow-up immediately after diagnosis were also excluded (*n* = 4), leaving 1476 women in the analysis.

### Determinants of ASP

Sociodemographic characteristics, tumour characteristics and type of treatment received, regardless of whether treatment was completed or not, were assessed in relation to each ASP self-reporting. These included: (1) Five population groups defined by country and ethnicity (Namibia black, Namibia non-black, Uganda, Nigeria and Zambia); (2) TNM stage at diagnosis (stage I/II, III, IV and unknown); (3) age at diagnosis (continuous, < 50 years and ≥ 50); (4) highest educational level (none or primary school, secondary or high school, university or technical degree); (5) body mass index (BMI) calculated from measured height and weight at baseline (continuous and < 25 kg/m^2^, [25–30[ Kg/m^2^, ≥ 30 kg/m^2^); (6) self-reported HIV status at baseline (positive, negative or unknown); (7) Self-reported hypertension (yes, no) at baseline based on the question “Have you ever been diagnosed with hypertension?”; (8) treatment and specific types of treatment (surgery, radiotherapy, chemotherapy, endocrine therapy) were considered as categorical time-varying variable, and we performed a Lexis expansion to take into account change in treatment status over time (prior treatment: yes/no/unknown) [20].

### Statistical analysis

For each ASP, we examined time to the first report of the ASP. Follow-up started from the date of diagnosis and ended on the date of interview when the ASP was first reported, the date of death (as a competing event), the date when 4-year follow-up was reached, date of last live contact or 1st January 2020, whichever came first. Using this time scale, we calculated the cumulative incidence of each ASP, overall and stratified by study site, from time since diagnosis prior to receiving treatment.

To identify potential determinants for each ASP, we fitted Cox proportional hazards models. Crude models were fitted for all potential determinants adjusted for age as a continuous variable and stratified by study site. We used likelihood ratio tests to determine whether age, BMI, educational level and tumour stage would be better explained as continuous or categorical. For each ASP, a multivariate model was fitted mutually adjusting for the same set of covariates (i.e. age, BMI, educational level, stage at diagnosis and treatment received). These models yielded adjusted cause-specific hazard ratios (CHR) estimates which shows the relative change in the rate of ASP according to each determinant, in women who are currently alive.

To assess the robustness of the findings we conducted further analysis to estimate: (1) cumulative incidence of the first self-reported ASP stratified by type of ASP and tumour stage at diagnosis; (2) cumulative incidence using a more strict definition of ASP based on time to the first report of a specific ASP in a 12-month period when there were multiple reports of that ASP (not necessarily consecutive); for the latter analyses, cumulative incidences are reported up to three years after diagnosis to allow for a subsequent multiple report within the following 12-months (maximal follow-up 4-years). In addition, we fitted the multivariate models described above but conditional on women having survived the first 6 months after diagnosis and excluding those with metastatic disease, as these women might not have received the same treatment as less advanced cases, and might have been at higher risk of reporting ASP. Lastly, to better understand the respective impacts of breast cancer itself and treatment on occurrence of ASP, we estimated one-year cumulative incidences of self-reported ASP prior to and after starting treatment. All analyses were performed using STATA v15.1.

## Results

### Study population

Of the 1476 women included in this analysis, there were 477 Namibian, 418 Ugandan, 383 Nigerian and 198 Zambian women. Table 1 presents baseline characteristics of these women. Briefly, mean age at diagnosis was 50.3 years (SD = 13.7). About half of the women had none or primary school education level (*N* = 657, 44.5%), and most were diagnosed with late-stage breast cancer (*N* = 1106, 75.0%). Concerning comorbidities, 342 (23.2%) of women were obese, 144 (9.8%) were HIV-positive and 426 (28.9%) had hypertension. Although there were important between-country disparities in treatment received, overall, 171 women (11.6%) did not receive any treatment, 928 (62.9%) received chemotherapy, 841 (57.0%) had surgery, 694 (47.0%) endocrine therapy, and 465 (31.5%) radiotherapy.

**Table 1:** Baseline characteristics of the newly diagnosed breast cancer patients enrolled in the ABC-DO cohort study

	Namibia Non-Black (*N* = 97)	Namibia Black (*N* = 380)	Uganda (*N* = 418)	Nigeria (*N* = 383)	Zambia (*N* = 198)	Overall (*N* = 1476)
	*N* (%)	*N* (%)	*N* (%)	*N* (%)	*N* (%)	*N* (%)
Mean age at diagnosis, years (SD)	56.7 (12.5)	52.6 (15.0)	48.4 (12.7)	48.7 (12.3)	50.0 (14.8)	50.3 (13.7)
*BMI, kg/m*^*2*^						
< 25	31 (32.0)	162 (42.6)	201 (48.1)	162 (42.3)	81 (40.9)	637 (43.2)
[25–30[	20 (20.6)	98 (25.8)	148 (35.4)	107 (27.9)	53 (26.8)	426 (28.9)
30 +	41 (42.3)	102 (26.8)	60 (14.4)	94 (24.5)	45 (22.7)	342 (23.2)
Unknown	5 (5.2)	18 (4.7)	9 (2.2)	20 (5.2)	19 (9.6)	71 (4.8)
*Education*						
None/primary	13 (13.4)	197 (51.8)	242 (57.9)	102 (26.6)	103 (52.0)	657 (44.5)
Secondary/high school	46 (47.4)	132 (34.7)	126 (30.1)	144 (37.6)	54 (27.3)	502 (34.0)
Technical/university	38 (39.2)	51 (13.4)	50 (12.0)	137 (35.8)	41 (20.7)	317 (21.5)
*HIV status*						
Positive	3 (3.1)	53 (13.9)	48 (11.5)	9 (2.3)	31 (15.7)	144 (9.8)
*Hypertension*						
Yes	49 (50.5)	155 (40.8)	65 (15.6)	100 (26.1)	57 (28.8)	426 (28.9)
*Tumour stage at diagnosis*						
Localized (stage TNM I/II)	48 (49.5)	73 (19.2)	96 (23.0)	39 (10.2)	19 (9.6)	275 (18.6)
Locally advanced (stage TNM III)	43 (44.3)	247 (65.0)	226 (54.1)	256 (66.8)	131 (66.2)	903 (61.2)
Metastatic (stage TNM IV)	6 (6.2)	60 (15.8)	64 (15.3)	60 (15.7)	13 (6.6)	203 (13.8)
Unknown	0 (0.0)	0 (0.0)	32 (7.7)	28 (7.3)	35 (17.7)	95 (6.4)
*Treated*						
No	0 (0.0)	1 (0.3)	36 (8.6)	95 (24.8)	39 (19.7)	171 (11.6)
Yes	97 (100.0)	376 (98.9)	359 (85.9)	262 (68.4)	140 (70.7)	1234 (83.6)
Unknown	0 (0.0)	3 (0.8)	23 (5.5)	26 (6.8)	19 (9.6)	71 (4.8)
*Surgery*^a^						
No	9 (9.3)	88 (23.2)	125 (29.9)	150 (39.2)	85 (42.9)	457 (31.0)
Yes	84 (86.6)	244 (64.2)	240 (57.4)	183 (47.8)	90 (45.5)	841 (57.0)
Unknown	4 (4.1)	48 (12.6)	53 (12.7)	50 (13.1)	23 (11.6)	178 (12.1)
*Radiotherapy*						
No	24 (24.7)	72 (18.9)	276 (66.0)	308 (80.4)	130 (65.7)	810 (54.9)
Yes	71 (73.2)	287 (75.5)	56 (13.4)	11 (2.9)	40 (20.2)	465 (31.5)
Unknown	0 (0.0)	0 (0.0)	0 (0.0)	0 (0.0)	0 (0.0)	0 (0.0)
*Endocrine therapy*						
No	18 (18.6)	76 (20.0)	194 (46.4)	210 (54.8)	109 (55.1)	607 (41.1)
Yes	76 (78.4)	276 (72.6)	151 (36.1)	127 (33.2)	64 (32.3)	694 (47.0)
Unknown	0 (0.0)	0 (0.0)	0 (0.0)	0 (0.0)	0 (0.0)	0 (0.0)
*Chemotherapy*						
No	26 (26.8)	64 (16.8)	74 (17.7)	164 (42.8)	55 (27.8)	383 (25.9)
Yes	70 (72.2)	290 (76.3)	283 (67.7)	170 (44.4)	115 (58.1)	928 (62.9)
Unknown	1 (1.0)	26 (6.8)	61 (14.6)	49 (12.8)	28 (14.1)	165 (11.2)

^a^In ABC-DO, surgeries were mostly mastectomies (82%) as compared to lumpectomies. These were, respectively, 80%, 92%, 91%, 50% and 94% in Namibian Non-Black, Namibian Black, Ugandan, Nigerian, and Zambian women

### Distribution of self-reported ASP

**Fig. 1:**
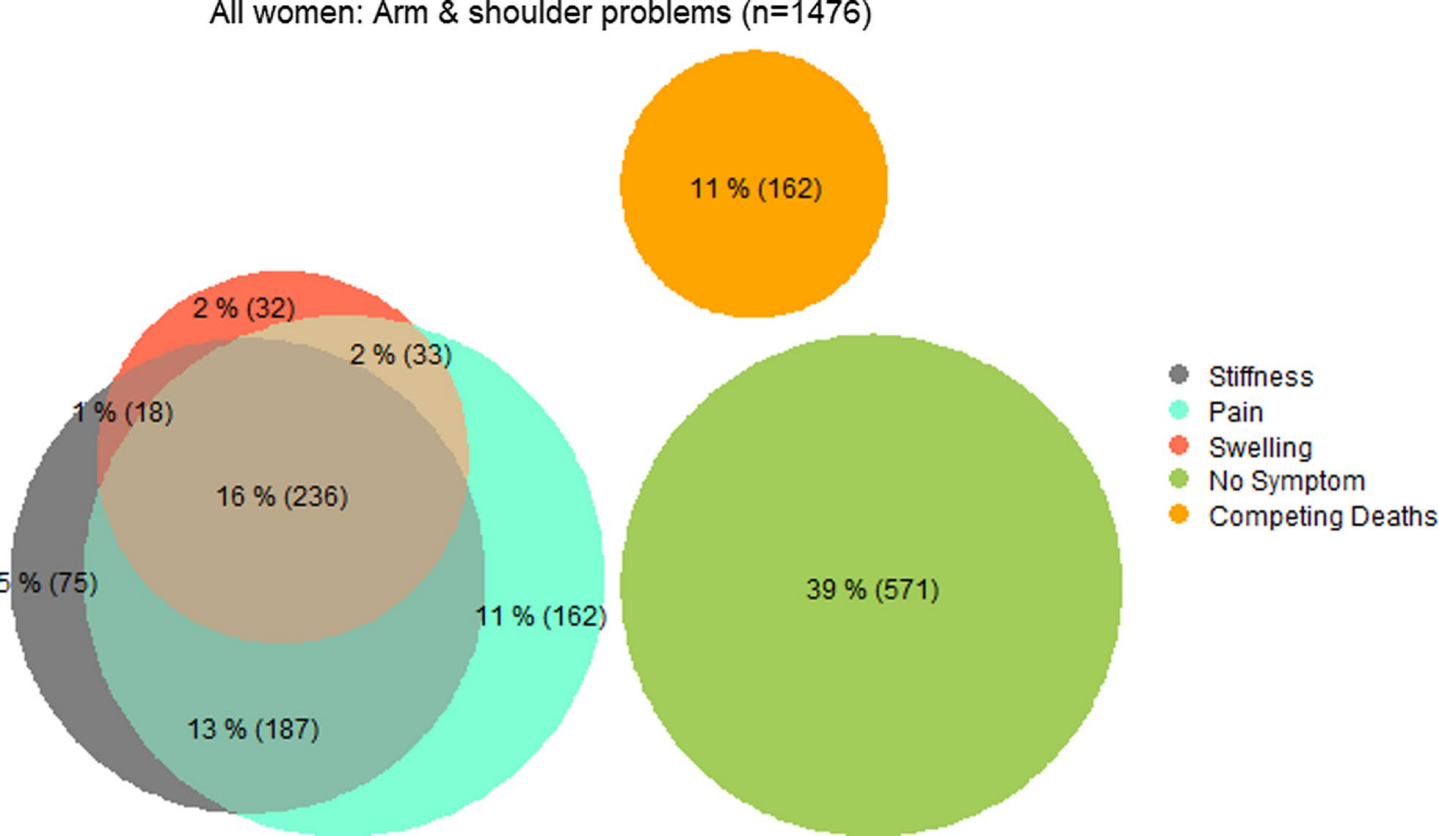
Crude proportion (*n* = 1476) of women who self-reported at least once having experienced ASP, by type of ASP, in ABC-DO

Of the 1476 women included in this analysis, 162 (11.0%) died before completing the first follow-up interview. Overall, 743 (50.3%) of the 1476 women reported at least once an ASP, 618 (41.9%) arm/shoulder pain, 516 (35.0%) arm stiffness and 319 (21.6%) arm/hand swelling (Fig. 1). Of the 238 (16.1%) women who reported having experienced two different ASP, either concomitantly or at different follow-up time points, 187 (78.6%) reported both arm/shoulder pain and arm stiffness, 33 (13.9%) reported pain and arm/hand swelling, and 18 (7.6%) reported both arm stiffness and swelling. Finally, 236 (16.0%) women reported having experienced all three ASP.

### Cumulative incidence of self-reported ASP

Four years after being diagnosed with breast cancer, cumulative incidences of shoulder/arm pain, stiffness and arm/hand swelling were 43.0% (95% CI 40.4–45.6), 35.8% (33.4–38.3) and 22.5% (20.4–24.8), based on 618, 516 and 319 women, respectively (Table 2). Among the 171 untreated women, these cumulative incidences were, respectively, 29.2% (21.9–36.8), 21.2% (14.7–28.4) and 15.3% (9.8–21.9) based on 47, 33 and 23 women, respectively (Additional file 1: Table S1). In comparison to prior to receiving any treatment, cumulative incidences of ASP were two to three times higher after treatment start (Additional file 2: Table S2 and Additional file 3: Fig. S1). About half of all women who reported a specific ASP had multiple reports of that ASP within a 12-months period (Additional file 4: Fig. S2). The rate at which new ASPs were reported declined over time, with half of the 4-year cumulative reporting incidence occurring within the first year of diagnosis (Additional file 4: Fig. S2).

**Table 2:** Four years post-diagnosis cumulative incidence of first self-reported ASP in ABCDO, by country and treatment

	No. women with outcome/total (competing deaths)	Time at risk (person-years)	ASP type-specific cumulative incidence at 4 years since diagnosis (95% CI)
Shoulder/arm pain			
All sites/ethnicities	618/1476 (449)	680	43.0 (40.4–45.6)
Namibia non-black	25/97 (12)	42	26.0 (17.7–35.1)
Namibia black	151/380 (100)	218	40.3 (35.3–45.2)
Uganda	214/418 (119)	174	51.8 (46.9–56.5)
Nigeria	162/383 (141)	168	44.0 (38.7–49.3)
Zambia	66/198 (77)	77	35.1 (28.3–42.0)
Arm stiffness			
All sites/ethnicities	516/1476 (505)	628	35.8 (33.4–38.3)
Namibia non-black	26/97 (13)	34	26.9 (18.5–36.0)
Namibia black	148/380 (106)	209	39.3 (34.3–44.2)
Uganda	184/418 (133)	201	44.7 (39.8–49.5)
Nigeria	106/383 (176)	124	28.8 (24.2–33.6)
Zambia	52/198 (77)	60	27.9 (21.6–34.5)
Arm/hand swelling			
All sites/ethnicities	319/1476 (602)	464	22.5 (20.4–24.8)
Namibia non-black	13/97 (16)	29	13.7 (7.7–21.4)
Namibia black	81/380 (130)	144	21.9 (17.8–26.3)
Uganda	103/418 (180)	122	25.1 (21.0–29.4)
Nigeria	84/383 (191)	120	23.3 (19.0–28.0)
Zambia	38/198 (85)	50	20.4 (15.0–26.5)

*ASP* arm and shoulder problems, *CI* confidence interval

### Determinants of first self-reported ASP

Determinants of first self-reported ASP, separately for each ASP type, are described in detail in Table 3, and in Additional file 5: Table S3 and Additional file 6: Fig. S3. After adjusting on all potential determinants of ASP identified in crude analysis, there were important between-country and between-ethnicity disparities. Relative to Namibia black women, the risk of a first ASP self-report was lowest for non-black Namibians for all three ASP and highest for Ugandan women for all ASP (fully adjusted CHR 2.00; 95%CI 1.61–2.48 for shoulder/arm pain, 1.52 (1.21–1.90) for arm stiffness and 1.59 (1.18–2.15) for arm/hand swelling), and for Nigerian women for all ASP except arm stiffness.

**Table 3:** Fully adjusted associations of baseline sociodemographic, tumour and treatment characteristics with first self-reported ASP

	No. women with outcome/total			Fully adjusted CHR (95%CI)^a,b^					
	Shoulder/arm pain	Arm stiffness	Arm/hand swelling	Shoulder/arm pain		Arm stiffness		Arm/hand swelling	
*Study site, ethnicity*									
Namibia Black	151/380	148/380	81/380	1		1		1	0.001
Namibia Non-Black	25/97	26/97	13/97	0.71 (0.46–1.10)	< 0.0001	0.78 (0.50–1.20)	0.0002	0.68 (0.37–1.24)	
Uganda	214/418	184/418	103/418	2.00 (1.61–2.48)		1.52 (1.21–1.90)		1.59 (1.18–2.15)	
Nigeria	162/383	106/383	84/383	1.88 (1.48–2.39)		1.06 (0.81–1.39)		1.69 (1.21–2.34)	
Zambia	66/198	52/198	38/198	1.19 (0.88–1.61)		0.91 (0.66–1.27)		1.35 (0.90–2.03)	
*Age at diagnosis (years)*									
< 50	307/780	255/780	157/780	1	0.06	1	0.23	1	0.10
≥ 50	311/696	261/696	162/696	1.18 (0.99–1.39)		1.12 (0.93–1.35)		1.22 (0.96–1.53)	
per 10 years increase	618/1476	516/1476	319/1476	1.04 (0.98–1.11)	0.21	1.04 (0.97–1.11)	0.31	1.04 (0.95–1.13)	0.43
*BMI (Kg/m*^*2*^*)*									
< 25	273/637	229/637	129/637	1	0.37	1	0.30	1	0.41
[25–30[	176/426	144/426	95/426	0.88 (0.72–1.06)		0.89 (0.72–1.09)		1.04 (0.79–1.35)	
30 +	140/342	120/342	80/342	0.99 (0.80–1.21)		1.07 (0.85–1.34)		1.21 (0.91–1.60)	
per 5 kg/m^2^ increase	618/1476	516/1476	319/1476	1.00 (0.93–1.07)	0.94	1.03 (0.96–1.12)	0.40	1.11 (1.01–1.22)	0.03
*Education*									
University/technical	106/317	84/317	66/317	1		1		1	0.07
Secondary/high school	196/502	161/502	95/502	1.48 (1.16–1.88)		1.41 (1.08–1.84)		1.08 (0.78–1.49)	
None/Primary school	316/657	271/657	158/657	1.94 (1.51–2.49)		1.77 (1.34–2.33)		1.41 (1.02–1.96)	
Per decrease in educational level	618/1476	516/1476	319/1476	1.38 (1.22–1.56)	< 0.0001	1.32 (1.16–1.51)	< 0.0001	1.20 (1.02–1.41)	0.03
*HIV status at breast cancer diagnosis*									
Negative/Unknown	562/1332	460/1332	291/1332	1	0.85	1	0.14	1	> 0.99
Positive	56/144	56/144	28/144	0.97 (0.73–1.29)		1.24 (0.93–1.65)		1.00 (0.67–1.49)	
*Ever diagnosed with hypertension*									
No	432/1050	363/1050	215/1050	1	0.11	1	0.99	1	0.15
Yes	186/426	153/426	104/426	1.17 (0.96–1.42)		1.00 (0.81–1.23)		1.22 (0.93–1.58)	
*Tumour stage at diagnosis*									
Localized	91/275	79/275	40/275	1	< 0.0001	1	< 0.0001	1	< 0.0001
Locally advanced	413/903	343/903	207/903	1.70 (1.35–2.14)		1.66 (1.29–2.13)		1.98 (1.40–2.79)	
Metastatic	79/203	68/203	48/203	2.44 (1.78–3.33)		2.69 (1.92–3.77)		4.02 (2.59–6.23)	
*Prior treatment*									
No	105/237	75/221	41/197	1	0.01	1	0.08	1	0.01
Yes	496/1168	429/1184	270/1208	1.37 (1.08–1.72)		1.27 (0.97–1.67)		1.67 (1.16–2.41)	
*Prior surgery*^c^									
No	248/512	185/497	116/482	1	0.01	1	0.21	1	0.19
Yes	309/786	280/801	173/816	0.76 (0.63–0.92)		0.87 (0.70–1.08)		0.83 (0.63–1.09)	
*Prior radiotherapy*									
No	397/854	316/850	186/825	1	0.60	1	0.22	1	0.59
Yes	147/421	135/425	86/450	1.07 (0.83–1.38)		0.85 (0.65–1.10)		1.10 (0.77–1.59)	
*Prior chemotherapy*									
No	184/438	133/418	79/400	1	0.0002	1	0.001	1	0.001
Yes	370/873	327/893	206/911	1.48 (1.21–1.82)		1.48 (1.17–1.87)		1.65 (1.22–2.23)	
*Prior endocrine therapy*									
No	328/703	247/672	151/649	1	0.59	1	0.87	1	0.45
Yes	234/598	218/629	132/652	1.06 (0.86–1.29)		0.98 (0.79–1.22)		0.90 (0.68–1.18)	
Sensitivity analysis conditioned on 6 months survival and excluding metastatic women									
*Prior treatment*									
Yes vs. No	419/1037	366/1039	214/1045	1.58 (1.14–2.18)	0.01	1.37 (0.95–1.98)	0.10	1.39 (0.86–2.24)	0.18
*Prior surgery*									
Yes vs. No	280/769	254/771	149/776	0.77 (0.62–0.95)	0.02	0.92 (0.73–1.17)	0.50	0.84 (0.62–1.14)	0.26
*Prior radiotherapy*									
Yes vs. No	125/382	116/380	76/401	0.83 (0.64–1.09)	0.18	0.70 (0.53–0.92)	0.01	0.91 (0.62–1.32)	0.61
*Prior chemotherapy*									
Yes vs. No	339/813	300/813	178/821	1.67 (1.31–2.12)	< 0.0001	1.52 (1.17–1.97)	0.002	1.59 (1.12–2.25)	0.01
*Prior endocrine therapy*									
Yes vs. No	218/576	200/582	118/597	0.97 (0.79–1.19)	0.74	0.99 (0.80–1.23)	0.93	0.87 (0.65–1.15)	0.32

*CHR* cause-specific hazard ratio, *CI* confidence interval

^a^For study site, age, BMI, education, tumour stage and prior treatment: fully adjusted CHRs are stratified on study site, and adjusted on age (continuous), BMI (categorical), education (continuous), tumour stage (categorical), and prior treatment

^b^For Prior surgery, prior radiotherapy, prior chemotherapy, Prior endocrine therapy: fully adjusted CHRs are stratified on study site, and adjusted on age (continuous), BMI (categorical), education (continuous), tumour stage (categorical), and mutually adjusted on each specific treatment type

^c^Of women who received a surgery and reported an arm/shoulder pain during the follow-up, 84% had a mastectomy. For arm stiffness and arm/hand swelling, these percentages were, respectively, 89% and 87%

Tumour stage at diagnosis was the main determinant of ASP self-reporting (Table 3 and Additional file 7: Fig. S4), with women diagnosed with a more advanced stage tumour having an increased risk of self-reporting an ASP (*p* for heterogeneity < 0.0001 for all three types of ASP). The strongest association was observed for arm swelling – relative to women with localized disease at diagnosis, the risk of self-reporting this ASP was two times higher (CHR 1.98; 95% CI 1.40–2.79) for women with locally advanced cancers, and four times higher (4.02; 2.59–6.23) for those with metastatic disease.

The risk of self-reporting an ASP was inversely associated with the woman’s educational level for all ASP (Table 3), being particularly marked for shoulder/arm pain and arm stiffness (*p* for trend < 0.0001). Relative to women with a university level, those with only primary school level or less were 94% (fully adjusted CHR 1.94; 95% CI 1.51–2.49) more likely to self-report shoulder/arm pain and 77% (1.77; 1.34–2.33) more likely to self-report arm stiffness. Older age at breast cancer diagnosis (≥ 50 years) tended to be associated with a higher reporting of shoulder/arm pain (CHR 1.18; 95% CI 0.99–1.39, *p* = 0.06), but there was no evidence that the risk of self-reporting any of the three ASPs depended on a woman’s BMI, HIV status or hypertension.

Receiving treatment was associated with the likelihood of reporting shoulder/arm pain (CHR 1.37; 95% CI 1.08–1.72) and arm/hand swelling (CHR 1.67; 95% CI 1.16–2.41). These associations were driven by chemotherapy, which increased the risk of reporting an ASP by about 50% for both shoulder/arm pain (CHR 1.48; 95% CI 1.21–1.82) and arm stiffness (1.48; 1.17–1.87), and by up to 65% for arm/hand swelling (1.65; 1.22–2.23). In contrast, surgery decreased the risk of reporting a shoulder/arm pain by about 25% (0.76; 0.63–0.92). However, when stratifying the analysis according to tumour stage at diagnosis, these treatment effects were only observed in women with late stage cancers (Additional file 8: Table S4).

After excluding women with metastatic cancer and conditioning the analysis on 6-month survival, results remained similar. However, receiving radiotherapy reduced the risk of reporting an arm stiffness, and this association was strongest in women diagnosed with a localized breast cancer (Additional file 8: Table S4).

## Discussion

### Main findings

Using data from a large and multi-centric cohort, we obtained robust estimates of the frequency of ASP in women after a breast cancer diagnosis across multiple SSA settings and examined their determinants. To our knowledge, our study is the first in SSA to show that ASP occur not only after receiving treatment but may also be present prior to treatment. The frequency of self-reported ASP was high in this setting, but with important between-country disparities. Overall, about 1 out of 2 women reported having experienced a moderate to severe ASP at least once during the follow-up period. Shoulder/arm pain was the most commonly reported ASP, followed by arm stiffness and arm/hand swelling, and most often, women reported multiple ASP types either concomitantly or at different follow-up time points. Among women who reported having experienced ASP, about half reported the same type of ASP more than once over time. More advanced breast cancer stage at diagnosis, older age, having a lower socioeconomic position and receiving treatment increased the risk of self-reported ASP.

### ASP frequency in SSA

Our estimates of ASP frequency in SSA were higher than those previously reported in the region, maybe due to differences in study designs, sample sizes, ASP assessment methods and follow-up durations [4, 9, 10]. There were, however, important between-country disparities in ASP reporting, with Namibian non-black women reporting ASP the least, and Ugandan women the most. These differences, which remained after controlling for the earlier stage at breast cancer diagnosis of non-Namibian black women, may reflect over- or under-reporting of ASP for cultural reasons, variations in data collection quality or in interpretation of EORTC-QLQ-Br23 questions across sites, and/or ethnic differences in a woman’s susceptibility to develop an ASP, because within Namibia, non-black women tended to report less ASP than black women. Lifestyle and treatment management and aftercare will also differ substantially.

### Sociodemographic determinants of ASP

In ABC-DO, women with lower educational level were at higher risk of self-reporting an ASP, irrespective of tumour stage at diagnosis, maybe due to lower breast cancer awareness and higher physical demands of their daily cores [11]. Women over 50 years of age tended to be at higher risk of self-reporting a shoulder/arm pain, which contrasts with the findings from a South African study in which the prevalence of ASP decreased with age [4]. Also, we did not find an association between BMI and ASP, possibly because it was only measured once, at breast cancer diagnosis, and we were not able to capture its change over time in the analysis [21].

### Medical determinants of ASP

In our study, a higher breast cancer stage at diagnosis was the most important determinant of self-reporting an ASP. In contrast to HICs where most women have an early stage diagnosis, late stage diagnosis for breast cancer is common in SSA due to low breast cancer awareness among both women and healthcare professionals, and long delays to presentation of symptomatic women to a healthcare provider, final diagnosis and treatment initiation, with disadvantaged populations being particularly affected [11, 12, 22, 23]. In our cohort, three quarters of women had a late stage breast cancer at diagnosis, with nearly half having a tumour size over five centimetres and about two thirds positive lymph nodes at diagnosis. It is plausible that, the larger tumours, and the increased number of affected lymph nodes in women with an advanced breast cancer, may have favoured ASP development, including prior to receiving treatment [24, 25].

Receiving treatment also increased the risk of reporting an ASP in women with advanced breast cancers, but our study may have lacked statistical power to detect an association in women with localized breast cancers. This association may partly be due to remaining unmeasured systematic differences between women who underwent specific treatment types and those who did not. The treatment effect was driven by chemotherapy, while surgery was associated with lower self-reporting of shoulder/arm pain, in line with what was found in another South African study with similar cohort characteristics [4]. Indeed, in our study, chemotherapy was the first treatment given and its initiation may have preceded the first follow-up interview. Women who received chemotherapy had higher SEP indicators and higher stage at diagnosis than women who did not. While chemotherapy is usually administered through central lines in HICs, these are not available in most LMICs where peripheral intravenous perfusions are used instead, as in our cohort. It is therefore possible that chemotherapy drugs administered through a peripheral line engender side-effects such as local inflammation and ASP. However, our study was not designed to assess treatment effects and thus we cannot exclude the possibility that the observed chemotherapy-ASP association may be due to local symptoms caused by the breast cancer itself [26, 27]. Despite the more invasive surgical procedures often used in SSA, as compared to HICs, such as mastectomy and axillary lymph node dissection, it is possible that surgical removal of large tumours or a high number of affected lymph nodes relieve pain in women with advanced disease [4, 8, 28, 29]. We also found that women diagnosed with an early stage cancer who survived at least six months had lower risk of self-reporting an ASP after receiving radiotherapy, which contrasts with previous studies results and needs to be further investigated [26].

### Strengths

To our knowledge, this study is the first to provide estimates of ASP burden related to breast cancer in Zambia, Uganda, and Namibia. Major strengths of this study were its multi-country design and large sample size; the use of a common protocol and data collection system for all four study sites, and of mHealth technology for study management, data collection and active follow-up of the participants. With the exception of Zambia, where an interruption of follow-up led to irreversible losses to follow-up, mHealth has ensured very few losses to follow-up in ABC-DO, and timely death notification [16]. The use of the validated EORTC-QLQ-Br23 questionnaire, a quality-of-life questionnaire with a special module for breast cancer patients, allowed us to capture accurate, affordable, and reproducible measures of ASP experienced by women in our cohort. Our study demonstrated that this questionnaire can be used to monitor breast cancer survivors across different countries and ethnicities in SSA, similarly to what has been shown in HICs [17]. Our large study population and relatively high number of arm and shoulder outcomes enabled us to obtain reliable and accurate estimates of ASP burden, and to study their determinants in the Sub-Saharan setting.

### Limitations

Our study population may not be representative of all breast cancer patients in SSA and the frequency of ASP. This is because recruitment was hospital-based, and some breast cancer cases may not seek care or may not be referred. However, hospital settings were tertiary centres, which is often the only treatment centre in the country and participation rate was very high (about 99%). After the baseline interview, at each 3-month follow-up contact, women were asked to report whether they had experienced ASP in the previous week. At the time of the first EORTC-QLQ-Br23 questionnaire, some women had already experienced an ASP. Therefore, our estimates are a proxy for incidence of ASP in this population because the baseline rates of ASP could not be determined in our population and those that occurred in between the 3-month follow-up interviews may have been missed. Moreover, EORTC-QLQ-Br23 questions did not specify the side affected by ASP. In light of these considerations, we would advise future studies focussed on ASPs to (1) perform clinical measurements whenever possible; (2) ascertain a time-stamped history of ASPs at the time of diagnosis; (3) for each of the above, separately ascertain ASPs for the affected and contralateral breast and, in parallel, obtain laterality information for all treatments administered. As compared to objective measurement, patients’ self-reports of arm swelling have been shown to have high sensitivity, but only moderate specificity [30], with the resulting false-positives leading to an over-estimation of the frequency of ASP. We improved specificity by only considering moderate to severe self-reported arm symptoms as indicative of ASP, as was done in other studies [5, 18, 19]. Moreover, the use of a validated questionnaire and standardized procedures to collect data on ASP limited the impact of misclassification on the study results. In our cohort, as the vast majority of women who received surgery got a mastectomy and data on axillary management was lacking, we were not able to analyse the impact of breast surgery type or axillary management technique on occurrence of ASP.

### Research implications

This study highlights the high frequency of ASP following a breast cancer diagnosis in SSA as well as the presence of marked differences between countries and, in Namibia, also between ethnic groups. Indeed, the cost of breast cancer-related ASP is not only physical or emotional by lowering a woman’s quality-of-life, but it may also have financial consequences, notably by impacting on a woman’s ability to work, or by engendering higher medical needs and costs (e.g. in the case of repeated infections due to lymphedema) [4, 31–33]. This may result in dramatic consequences on a woman’s ability to get appropriate care and take care of her family. Thus, identifying modifiable risk factors of ASP that could be targeted by future interventions is crucial to help preventing ASP in SSA. Further research is needed to better understand the impact of treatment on the occurrence of ASP in this setting (e.g. studies with complete treatment data including quality of surgery and after care, chemotherapy administration mode and associated side-effects, studies comparing women’s pain score pre- and post-surgery).

As WHO launches its Global Breast Cancer Initiative in 2021, improving the diagnosis, prevention, and management of ASP in LMICs is important. Breast cancer care and survivorship programmes need to be developed in SSA, and should combine educational, financial, and emotional support components to significantly improve breast cancer patient’s quality-of-life and reduce the burden of ASP [25, 34–36]. These programmes should address ASP awareness, prevention, detection, and management. Moreover, they would need to reach breast cancer patients, health care professionals and also traditional healers, as these play an important role in cancer care delivery in this setting [37]. Affordable self-management measures should be emphasized and could be implemented in settings where access to care is lacking, and women should have access to physical and decongestive therapies whenever possible. Moreover, downstaging breast cancer is crucial to further reduce ASP burden in SSA, by raising awareness of this disease and by promoting its early detection in the region, especially among underprivileged populations who are the most at risk of late breast cancer diagnosis.

## Conclusion

This study provides up-to-date estimates of ASP burden in women living with breast cancer in SSA and gives insights on their determinants. In this setting, women affected by breast cancer are at high risk of reporting an ASP that may significantly impair their quality-of-life and contribute to worsen social inequities. To reduce the burden of ASP, appropriate breast cancer downstaging strategies, as well as patient and survivorship care programmes that include long-term surveillance, are needed.

## Supplementary Information

**Additional file 1: Table S1.** Four years post-diagnosis cumulative incidence of first self-reported ASP in untreated women in ABC-DO.

**Additional file 2: Table S2.** One-year cumulative incidences of first self-reported ASP prior to and after starting treatment in ABC-DO.

**Additional file 3: Fig. S1.** Cumulative incidence curves of first self-reported ASP, prior to and after starting treatment in ABC-DO.

**Additional file 4: Fig. S2.** Cumulative incidence curve of first and multiple self-reported ASP, by country and ethnicity.

**Additional file 5: Table S3.** Crude associations of baseline sociodemographic, tumour and treatment characteristics with first self-reported ASP.

**Additional file 6: Fig. S3.** Forest plot of determinants of first self-reported ASP. This forest plot shows fully adjusted associations between baseline and tumour characteristics, and treatment types with first self-reports of each ASP.

**Additional file 7: Fig. S4.** Cumulative incidence curve of first self-reported ASP, by tumour stage.

**Additional file 8: Table S4.** Fully adjusted associations between treatment types and first self-reported ASP, by tumour stage.

## Data Availability

All data generated or analysed during this study are included in this published article and its supplementary information files.

## Abbreviations

ABC-DO: African Breast Cancer-Disparities in Outcomes
ASP: Arm and shoulder problems
BMI: Body Mass Index
CHR: Cause-specific Hazard ratio
CI: Confidence interval
EORTC-QLQ-BR23: European Organisation for Research and Treatment of Cancer breast-cancer specific quality-of-life questionnaire module
IARC: International Agency for Research on Cancer
HIC: High-income countries
IQR: Interquartile range
LMIC: Low- and middle-income countries
SD: Standard deviation
SEP: Socioeconomic position
SSA: Sub-Saharan Africa
WHO: World Health Organisation
