# Supplementary material for: Self-reported arm and shoulder problems in breast cancer survivors in Sub-Saharan Africa: the African Breast Cancer-Disparities in Outcomes cohort study
Source: Breast Cancer Res. 2021 Nov 24;23:109. doi: 10.1186/s13058-021-01486-9 (PMC8611842; doi:10.1186/s13058-021-01486-9)
Supplement: Supplementary file 1 — Additional file 1: Table S1. Four years post-diagnosis cumulative incidence of first self-reported ASP in untreated women in ABC-DO. [file 13058_2021_1486_MOESM1_ESM.docx]

Supplemental Table 1. Four years post-diagnosis cumulative incidence of first self-reported ASP in untreated women in ABC-DO

|  | **No. untreated women with outcome / total (competing deaths)** | **Time at risk (Person-Years)** | **ASP type-specific cumulative incidence at 4 years since diagnosis (95%CI)** |
| --- | --- | --- | --- |
| **Any shoulder/arm pain** | | | |
| All sites/ethnicities | 47/171 (84) | 36 | 29.2 (21.9-36.8) |
| **Any arm stiffness** | | | |
| All sites/ethnicities | 33/171 (93) | 36 | 21.2 (14.7-28.4) |
| **Any arm/hand swelling** | | | |
| All sites/ethnicities | 23/171 (101) | 32 | 15.3 (9.8-21.9) |
