# Supplementary material for: Self-reported arm and shoulder problems in breast cancer survivors in Sub-Saharan Africa: the African Breast Cancer-Disparities in Outcomes cohort study
Source: Breast Cancer Res. 2021 Nov 24;23:109. doi: 10.1186/s13058-021-01486-9 (PMC8611842; doi:10.1186/s13058-021-01486-9)
Supplement: Supplementary file 2 — Additional file 2: Table S2. One-year cumulative incidences of first self-reported ASP prior to and after starting treatment in ABC-DO. [file 13058_2021_1486_MOESM2_ESM.docx]

**Supplemental Table 2. One-year cumulative incidences of first self-reported ASP prior to and after starting treatment in ABC-DO**

|  | **Prior to receiving treatment** | | | **After treatment start** | | |
| --- | --- | --- | --- | --- | --- | --- |
|  | **No. women with outcome / total (competing deaths)** | **Time at risk (Person-Years)** | **ASP type-specific cumulative incidence at 1 year since diagnosis (95% CI)** | **No. women with outcome / total (competing deaths)** | **Time at risk (Person-Years)** | **ASP type-specific cumulative incidence at 1 year since first treatment (95% CI)** |
| **Shoulder/arm pain** | | | | | | |
| All sites/ethnicities | 25/1094(4) | 10 | 8.8(4.9-14.1) | 473/1113(291) | 475 | 25.8(23.3-28.5) |
| Namibia non-black | 0/88(0) | - | - | 25/95(12) | 39 | 10.5(5.4-17.6) |
| Namibia black | 0/349(1) | - | - | 149/369(94) | 186 | 21.4(17.4-25.7) |
| Uganda | 15/326(1) | 6 | 16.7(6.1-31.8) | 144/300(80) | 101 | 36.4(30.9-41.8) |
| Nigeria | 9/206(2) | 4 | 12.2(4.3-24.5) | 100/217(66) | 96 | 28.2(22.4-34.3) |
| Zambia | 1/125(0) | 0 | 1.0(0.1-5.0) | 55/132(39) | 53 | 21.4(14.8-28.8) |
| **Arm stiffness** | | | | | | |
| All sites/ethnicities | 23/1094(4) | 10 | 8.4(4.6-13.8) | 412/1131(332) | 456 | 19.0(16.7-21.3) |
| Namibia non-black | 0/88(0) | - | - | 26/95(13) | 30 | 14.7(8.5-22.6) |
| Namibia black | 0/349(1) | - | - | 146/369(100) | 180 | 19.5(15.6-23.7) |
| Uganda | 15/326(1) | 8 | 15.6(6.1-29.1) | 135/315(92) | 136 | 24.1(19.6-29.0) |
| Nigeria | 7/206(2) | 2 | 11.0(3.6-23.0) | 65/220(89) | 72 | 14.6(10.3-19.6) |
| Zambia | 1/125(0) | 0 | 1.0(0.1-4.7) | 40/132(38) | 38 | 15.3(9.8-22.1) |
| **Arm/hand swelling** | | | | | | |
| All sites/ethnicities | 13/1094(4) | 6 | 4.9(2.2-9.3) | 254/1154(414) | 338 | 9.5(7.9-11.2) |
| Namibia non-black | 0/88(0) | - | - | 13/95(16) | 26 | 2.1(0.4-6.7) |
| Namibia black | 0/349(1) | - | - | 79/369(123) | 125 | 7.0(4.7-10.0) |
| Uganda | 7/326(1) | 4 | 5.1(1.7-11.3) | 76/331(132) | 85 | 12.4(9.1-16.2) |
| Nigeria | 6/206(2) | 2 | 10.4(3.2-22.7) | 53/224(98) | 67 | 11.2(7.5-15.7) |
| Zambia | 0/125(0) | - | - | 33/135(45) | 36 | 11.2(6.6-17.3) |
