# Supplementary figures and images for: Self-reported arm and shoulder problems in breast cancer survivors in Sub-Saharan Africa: the African Breast Cancer-Disparities in Outcomes cohort study
Source: Breast Cancer Res. 2021 Nov 24;23:109. doi: 10.1186/s13058-021-01486-9 (PMC8611842; doi:10.1186/s13058-021-01486-9)

## Slide 1
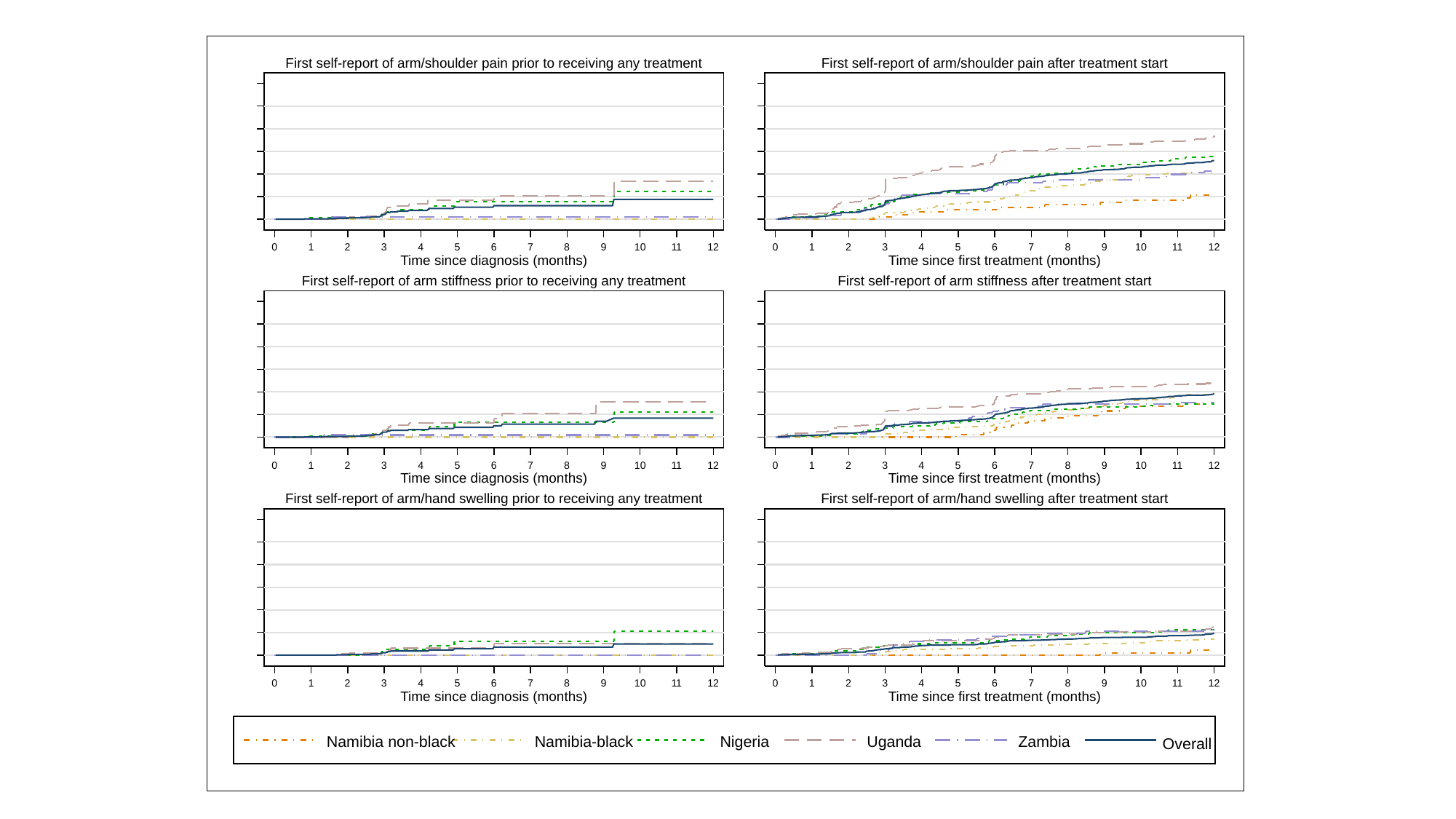

Supplement: Supplementary file 3 — Additional file 3: Fig. S1. Cumulative incidence curves of first self-reported ASP, prior to and after starting treatment in ABC-DO. [file 13058_2021_1486_MOESM3_ESM.pptx]

## Slide 1
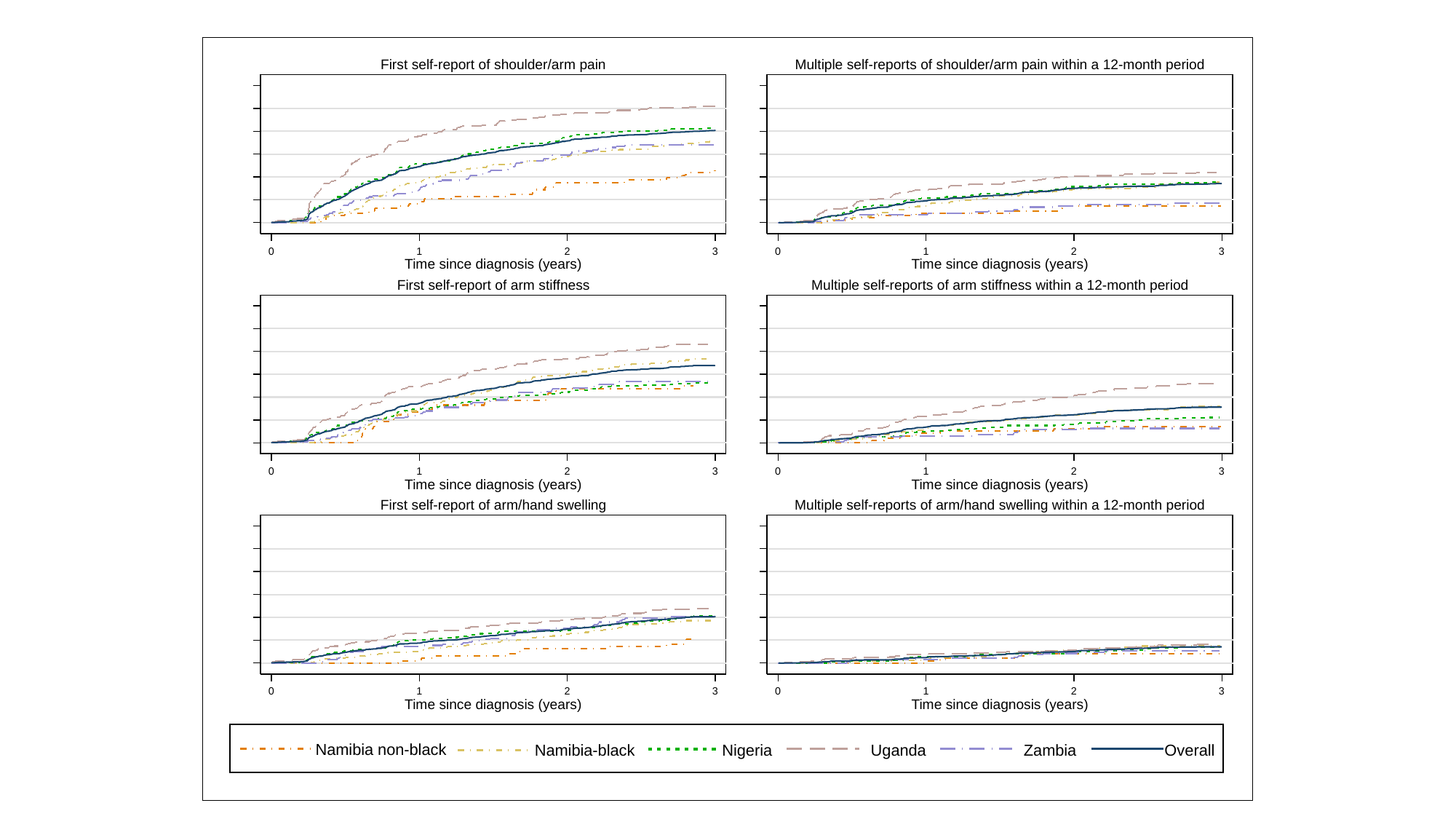

Supplement: Supplementary file 4 — Additional file 4: Fig. S2. Cumulative incidence curve of first and multiple self-reported ASP, by country and ethnicity. [file 13058_2021_1486_MOESM4_ESM.pptx]

## Slide 1
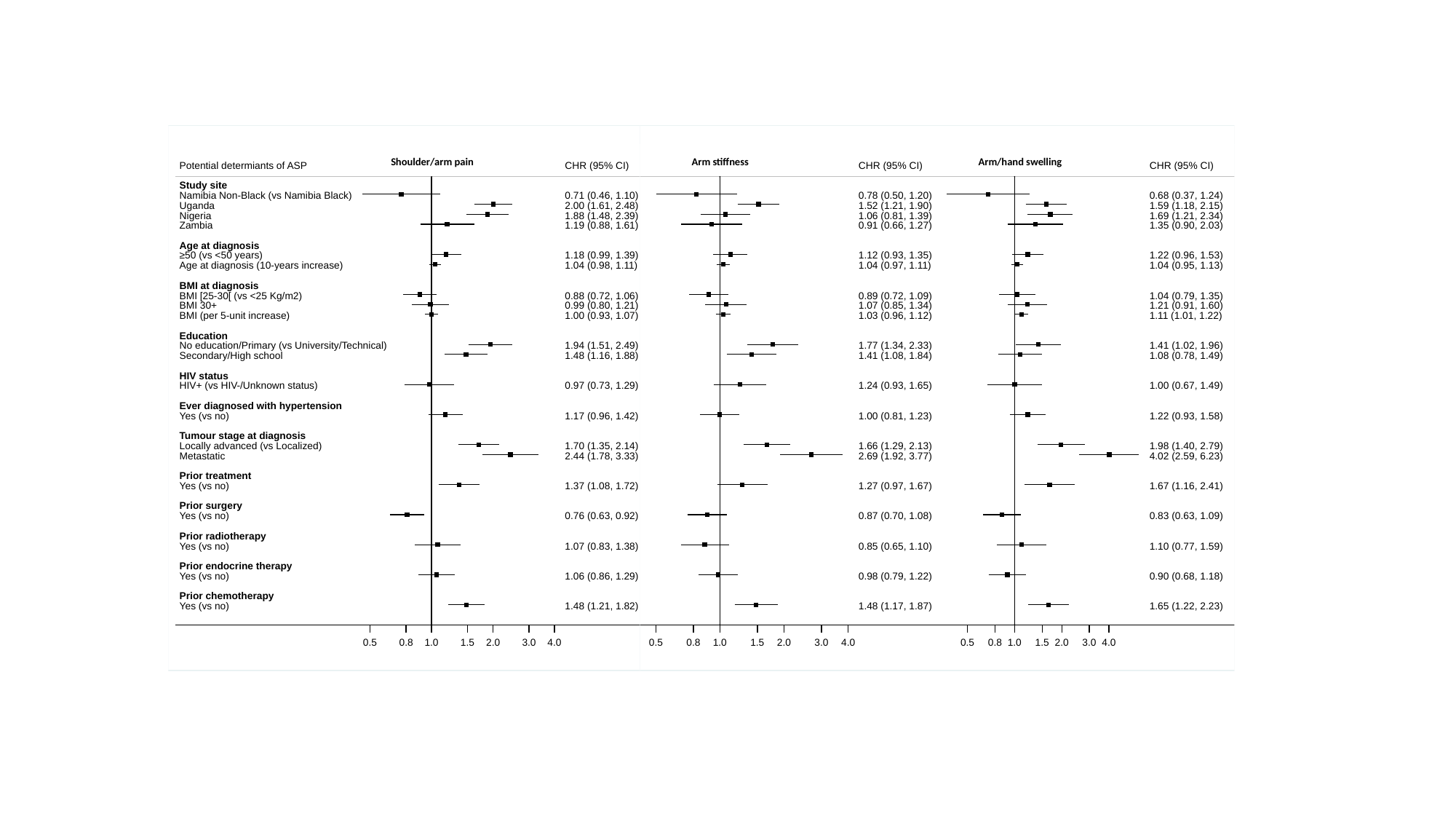

Arm stiffness
Shoulder/arm pain
Arm/hand swelling

Supplement: Supplementary file 6 — Additional file 6: Fig. S3. Forest plot of determinants of first self-reported ASP. This forest plot shows fully adjusted associations between baseline and tumour characteristics, and treatment types with first self-reports of each ASP. [file 13058_2021_1486_MOESM6_ESM.pptx]

## Slide 1
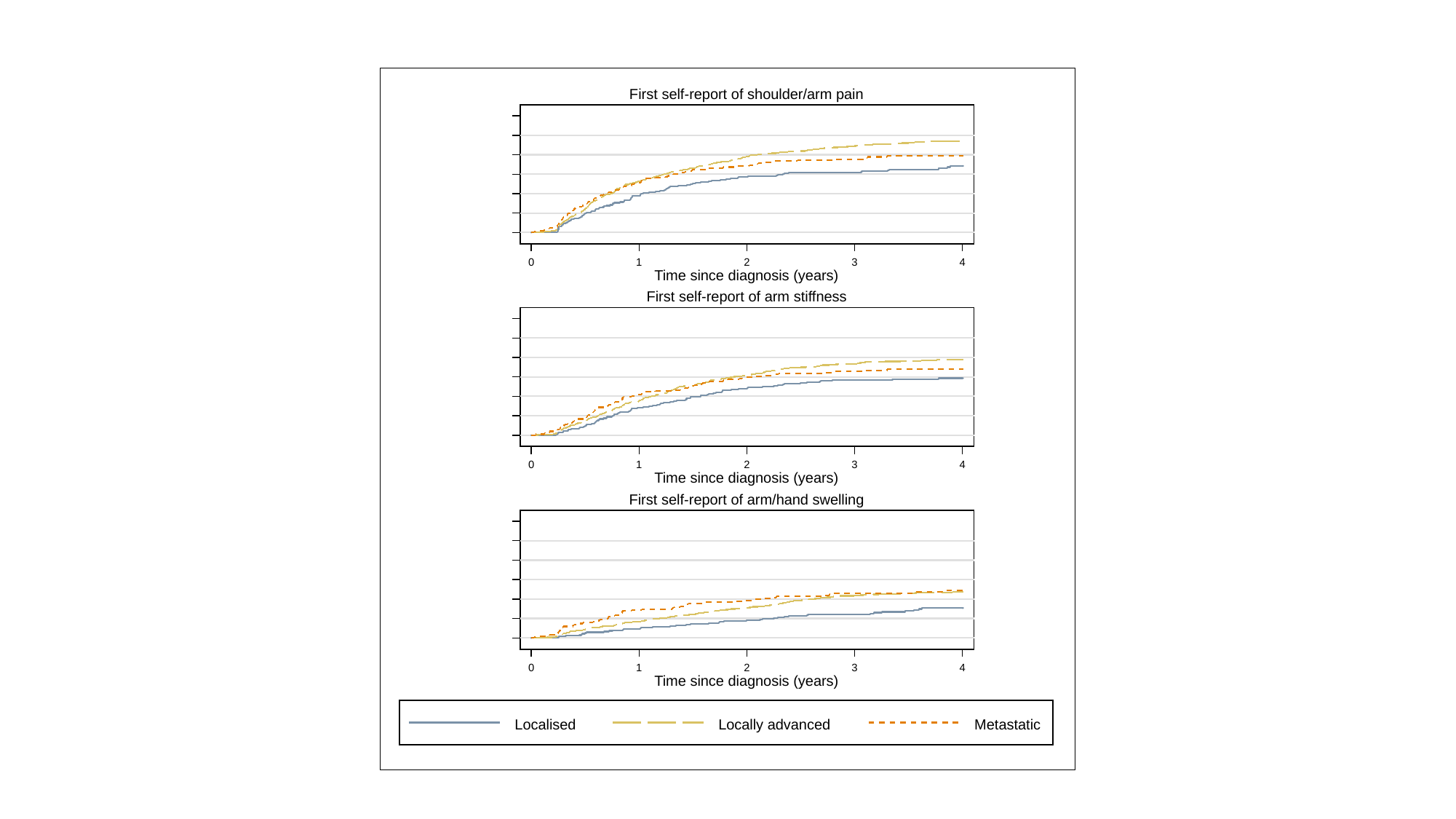

Supplement: Supplementary file 7 — Additional file 7: Fig. S4. Cumulative incidence curve of first self-reported ASP, by tumour stage. [file 13058_2021_1486_MOESM7_ESM.pptx]
