# Supplementary material for: Self-reported arm and shoulder problems in breast cancer survivors in Sub-Saharan Africa: the African Breast Cancer-Disparities in Outcomes cohort study
Source: Breast Cancer Res. 2021 Nov 24;23:109. doi: 10.1186/s13058-021-01486-9 (PMC8611842; doi:10.1186/s13058-021-01486-9)
Supplement: Supplementary file 5 — Additional file 5: Table S3. Crude associations of baseline sociodemographic, tumour and treatment characteristics with first self-reported ASP. [file 13058_2021_1486_MOESM5_ESM.docx]

Supplemental Table 3. Crude associations of baseline sociodemographic, tumour and treatment characteristics with first self-reported ASP

|  | | **No. women with outcome / total** | | | **Crude CHR (95%CI)*** | | | | | |
| --- | --- | --- | --- | --- | --- | --- | --- | --- | --- | --- |
|  |  | **Shoulder / arm pain** | **Arm stiffness** | **Arm / hand swelling** | **Shoulder/arm pain** | | **Arm stiffness** | | **Arm/hand swelling** | |
|  | **Namibia Black** | 151/380 | 148/380 | 81/380 | 1 |  | 1 |  | 1 | 0.001 |
| **Study site, ethnicity** | **Namibia Non-Black** | 25/97 | 26/97 | 13/97 | 0.48 (0.31-0.73) | <0.0001 | 0.53 (0.35-0.80) | <0.0001 | 0.49 (0.27-0.87) |  |
|  | **Uganda** | 214/418 | 184/418 | 103/418 | 1.86 (1.51-2.30) |  | 1.43 (1.15-1.78) |  | 1.46 (1.09-1.95) |  |
|  | **Nigeria** | 162/383 | 106/383 | 84/383 | 1.54 (1.23-1.92) |  | 0.90 (0.70-1.15) |  | 1.45 (1.07-1.98) |  |
|  | **Zambia** | 66/198 | 52/198 | 38/198 | 1.09 (0.81-1.46) |  | 0.83 (0.60-1.14) |  | 1.26 (0.86-1.86) |  |
| **Age at diagnosis (years)** | **<50** | 307/780 | 255/780 | 157/780 | 1 | 0.001 | 1 | 0.02 | 1 | 0.04 |
|  | **≥ 50** | 311/696 | 261/696 | 162/696 | 1.31 (1.11-1.53) |  | 1.23 (1.03-1.46) |  | 1.26 (1.01-1.57) |  |
|  | **per 10 years increase** | 618/1476 | 516/1476 | 319/1476 | 1.11 (1.04-1.18) | 0.001 | 1.09 (1.03-1.17) | 0.01 | 1.07 (0.98-1.16) | 0.11 |
| **BMI (Kg/m^2^)** | **<25** | 273/637 | 229/637 | 129/637 | 1 | 0.09 | 1 | 0.16 | 1 | 0.55 |
|  | **[25-30[** | 176/426 | 144/426 | 95/426 | 0.81 (0.67-0.98) |  | 0.83 (0.67-1.02) |  | 0.97 (0.74-1.26) |  |
|  | **30+** | 140/342 | 120/342 | 80/342 | 0.92 (0.75-1.13) |  | 1.00 (0.80-1.25) |  | 1.14 (0.86-1.51) |  |
|  | **per 5 Kg/m^2^ increase** | 618/1476 | 516/1476 | 319/1476 | 0.97 (0.90-1.04) | 0.42 | 1.01 (0.93-1.09) | 0.88 | 1.08 (0.98-1.19) | 0.10 |
|  | **University/technical** | 106/317 | 84/317 | 66/317 | 1 |  | 1 |  | 1 | 0.06 |
| **Education** | **Secondary/high school** | 196/502 | 161/502 | 95/502 | 1.46 (1.15-1.86) |  | 1.41 (1.08-1.84) |  | 1.09 (0.79-1.50) |  |
|  | **None/Primary school** | 316/657 | 271/657 | 158/657 | 2.00 (1.56-2.55) |  | 1.86 (1.42-2.43) |  | 1.42 (1.03-1.96) |  |
|  | **Per decrease in educational level** | 618/1476 | 516/1476 | 319/1476 | 1.41 (1.25-1.58) | <0.0001 | 1.36 (1.19-1.55) | <0.0001 | 1.20 (1.02-1.41) | 0.03 |
| **HIV status at breast cancer diagnosis** | **Negative/Unknown** | 562/1332 | 460/1332 | 291/1332 | 1 | 0.80 | 1 | 0.16 | 1 | 0.85 |
|  | **Positive** | 56/144 | 56/144 | 28/144 | 0.96 (0.73-1.28) |  | 1.22 (0.92-1.63) |  | 0.96 (0.65-1.43) |  |
| **Ever diagnosed with hypertension** | **No** | 432/1050 | 363/1050 | 215/1050 | 1 | 0.42 | 1 | 0.71 | 1 | 0.21 |
|  | **Yes** | 186/426 | 153/426 | 104/426 | 1.08 (0.89-1.31) |  | 0.96 (0.78-1.18) |  | 1.18 (0.91-1.53) |  |
| **Tumour stage at diagnosis** | **Localised** | 91/275 | 79/275 | 40/275 | 1 | <0.0001 | 1 | <0.0001 | 1 | <0.0001 |
|  | **Locally advanced** | 413/903 | 343/903 | 207/903 | 1.79 (1.42-2.26) |  | 1.75 (1.36-2.24) |  | 2.01 (1.43-2.84) |  |
|  | **Metastatic** | 79/203 | 68/203 | 48/203 | 2.68 (1.97-3.64) |  | 2.90 (2.08-4.04) |  | 4.21 (2.74-6.47) |  |
| **Prior treatment** | **No** | 105/237 | 75/221 | 41/197 | 1 | 0.01 | 1 | 0.08 | 1 | 0.01 |
|  | **Yes** | 496/1168 | 429/1184 | 270/1208 | 1.34 (1.06-1.68) |  | 1.27 (0.97-1.66) |  | 1.64 (1.14-2.36) |  |
| **Prior surgery** | **No** | 248/512 | 185/497 | 116/482 | 1 | 0.03 | 1 | 0.30 | 1 | 0.58 |
|  | **Yes** | 309/786 | 280/801 | 173/816 | 0.82 (0.68-0.97) |  | 0.90 (0.74-1.10) |  | 0.93 (0.72-1.20) |  |
| **Prior radiotherapy** | **No** | 397/854 | 316/850 | 186/825 | 1 | 0.35 | 1 | 0.41 | 1 | 0.46 |
|  | **Yes** | 147/421 | 135/425 | 86/450 | 1.12 (0.88-1.42) |  | 0.90 (0.71-1.15) |  | 1.13 (0.81-1.58) |  |
| **Prior chemotherapy** | **No** | 184/438 | 133/418 | 79/400 | 1 | 0.0004 | 1 | 0.001 | 1 | 0.0004 |
|  | **Yes** | 370/873 | 327/893 | 206/911 | 1.41 (1.17-1.70) |  | 1.45 (1.17-1.80) |  | 1.65 (1.25-2.17) |  |
| **Prior endocrine therapy** | **No** | 328/703 | 247/672 | 151/649 | 1 | 0.40 | 1 | 0.88 | 1 | 0.90 |
|  | **Yes** | 234/598 | 218/629 | 132/652 | 1.09 (0.90-1.31) |  | 1.02 (0.83-1.24) |  | 0.98 (0.76-1.28) |  |
| **Sensitivity analysis conditioned on 6 months survival and excluding metastatic women** | | | | | | | | | | |
| **Prior treatment** | **Yes vs. No** | 419/1037 | 366/1039 | 214/1045 | 1.54 (1.12-2.12) | 0.01 | 1.38 (0.96-1.98) | 0.09 | 1.36 (0.85-2.18) | 0.19 |
| **Prior surgery** | **Yes vs. No** | 280/769 | 254/771 | 149/776 | 0.77 (0.63-0.93) | 0.01 | 0.86 (0.69-1.07) | 0.17 | 0.82 (0.62-1.09) | 0.18 |
| **Prior radiotherapy** | **Yes vs. No** | 125/382 | 116/380 | 76/401 | 0.84 (0.65-1.07) | 0.16 | 0.74 (0.57-0.95) | 0.02 | 0.92 (0.65-1.30) | 0.64 |
| **Prior chemotherapy** | **Yes vs. No** | 339/813 | 300/813 | 178/821 | 1.53 (1.23-1.91) | 0.0001 | 1.47 (1.15-1.87) | 0.002 | 1.53 (1.11-2.10) | 0.01 |
| **Prior endocrine therapy** | **Yes vs. No** | 218/576 | 200/582 | 118/597 | 0.96 (0.79-1.18) | 0.72 | 0.96 (0.78-1.19) | 0.73 | 0.89 (0.67-1.17) | 0.40 |

CHR: Cause-specific hazard ratio; CI: Confidence interval

*CHR are presented adjusted on age (continuous), and stratified on study sites and ethnicity
