# Supplementary material for: Self-reported arm and shoulder problems in breast cancer survivors in Sub-Saharan Africa: the African Breast Cancer-Disparities in Outcomes cohort study
Source: Breast Cancer Res. 2021 Nov 24;23:109. doi: 10.1186/s13058-021-01486-9 (PMC8611842; doi:10.1186/s13058-021-01486-9)
Supplement: Supplementary file 8 — Additional file 8: Table S4. Fully adjusted associations between treatment types and first self-reported ASP, by tumour stage. [file 13058_2021_1486_MOESM8_ESM.docx]

Supplemental Table 4. Fully adjusted associations between treatment types and first self-reported ASP, by tumour stage

|  | | **No. women with outcome / total** | | | **Fully adjusted CHR (95%CI)*** | | | | | |
| --- | --- | --- | --- | --- | --- | --- | --- | --- | --- | --- |
|  |  | **Shoulder/arm pain** | **Arm stiffness** | **Arm/hand swelling** | **Shoulder/arm pain** | | **Arm stiffness** | | **Arm/hand swelling** | |
| **Prior treatment** | | | | | | | | | | |
| Early stage (stage I/II) | Yes vs. No | 76/241 | 69/242 | 36/248 | 1.06(0.57-1.99) | 0.85 | 0.94(0.44-1.98) | 0.87 | 0.98(0.31-3.11) | 0.97 |
| Late stage (stage III/IV) | Yes vs. No | 398/878 | 345/891 | 221/910 | 1.40(1.07-1.83) | 0.01 | 1.35(0.99-1.85) | 0.06 | 2.01(1.29-3.14) | 0.002 |
| **Prior surgery** | | | | | | | | | | |
| Early stage (stage I/II) | Yes vs. No | 57/200 | 55/204 | 28/210 | 0.78(0.47-1.28) | 0.32 | 0.96(0.55-1.67) | 0.89 | 0.53(0.24-1.18) | 0.12 |
| Late stage (stage III/IV) | Yes vs. No | 234/548 | 212/558 | 133/568 | 0.74(0.60-0.93) | 0.01 | 0.84(0.66-1.08) | 0.17 | 0.84(0.61-1.14) | 0.26 |
| **Prior radiotherapy** | | | | | | | | | | |
| Early stage (stage I/II) | Yes vs. No | 30/108 | 26/107 | 19/115 | 1.28(0.71-2.29) | 0.41 | 0.75(0.42-1.34) | 0.34 | 1.51(0.67-3.38) | 0.32 |
| Late stage (stage III/IV) | Yes vs. No | 114/308 | 108/314 | 64/330 | 1.03(0.77-1.39) | 0.83 | 0.87(0.64-1.17) | 0.35 | 0.96(0.63-1.47) | 0.85 |
| **Prior chemotherapy** | | | | | | | | | | |
| Early stage (stage I/II) | Yes vs. No | 50/159 | 51/164 | 30/168 | 1.21(0.74-2.00) | 0.44 | 1.44(0.81-2.55) | 0.21 | 1.93(0.86-4.35) | 0.11 |
| Late stage (stage III/IV) | Yes vs. No | 303/678 | 266/692 | 166/707 | 1.48(1.17-1.88) | 0.001 | 1.47(1.12-1.92) | 0.01 | 1.56(1.10-2.20) | 0.01 |
| **Prior endocrine therapy** | | | | | | | | | | |
| Early stage (stage I/II) | Yes vs. No | 35/140 | 36/147 | 19/155 | 0.85(0.49-1.49) | 0.58 | 0.97(0.56-1.70) | 0.93 | 0.79(0.37-1.67) | 0.54 |
| Late stage (stage III/IV) | Yes vs. No | 189/442 | 174/463 | 107/479 | 1.05(0.84-1.31) | 0.69 | 0.97(0.76-1.23) | 0.79 | 0.89(0.66-1.22) | 0.48 |
| **Sensitivity analysis conditioned on 6 months survival and excluding metastatic women** | | | | | | | | | | |
| **Prior treatment** | | | | | | | | | | |
| Early stage (stage I/II) | Yes vs. No | 68/247 | 68/247 | 31/248 | 0.84(0.37-1.93) | 0.69 | 1.06(0.41-2.74) | 0.90 | 0.46(0.13-1.67) | 0.24 |
| Late stage (stage III) | Yes vs. No | 329/735 | 283/737 | 172/742 | 1.54(1.06-2.23) | 0.02 | 1.38(0.90-2.14) | 0.14 | 1.63(0.91-2.93) | 0.10 |
| **Prior surgery** | | | | | | | | | | |
| Early stage (stage I/II) | Yes vs. No | 54/211 | 57/211 | 25/212 | 0.90(0.51-1.60) | 0.72 | 1.33(0.71-2.49) | 0.38 | 0.54(0.23-1.27) | 0.16 |
| Late stage (stage III) | Yes vs. No | 208/515 | 184/517 | 113/521 | 0.71(0.56-0.91) | 0.01 | 0.84(0.64-1.10) | 0.20 | 0.83(0.59-1.16) | 0.28 |
| **Prior radiotherapy** | | | | | | | | | | |
| Early stage (stage I/II) | Yes vs. No | 25/111 | 24/109 | 17/117 | 0.68(0.37-1.22) | 0.20 | 0.50(0.28-0.90) | 0.02 | 1.23(0.53-2.88) | 0.63 |
| Late stage (stage III) | Yes vs. No | 97/266 | 91/267 | 56/279 | 0.87(0.64-1.18) | 0.37 | 0.74(0.54-1.02) | 0.06 | 0.76(0.49-1.19) | 0.23 |
| **Prior chemotherapy** | | | | | | | | | | |
| Early stage (stage I/II) | Yes vs. No | 48/168 | 51/168 | 26/169 | 1.43(0.81-2.51) | 0.22 | 1.53(0.82-2.84) | 0.18 | 1.65(0.67-4.03) | 0.27 |
| Late stage (stage III) | Yes vs. No | 275/604 | 239/604 | 144/611 | 1.67(1.27-2.20) | 0.0003 | 1.49(1.10-2.02) | 0.01 | 1.58(1.06-2.36) | 0.02 |
| **Prior endocrine therapy** | | | | | | | | | | |
| Early stage (stage I/II) | Yes vs. No | 39/153 | 39/155 | 19/159 | 1.05(0.60-1.82) | 0.87 | 1.22(0.70-2.12) | 0.49 | 0.90(0.41-1.95) | 0.79 |
| Late stage (stage III) | Yes vs. No | 168/402 | 153/407 | 94/417 | 0.91(0.72-1.15) | 0.43 | 0.96(0.75-1.24) | 0.78 | 0.84(0.61-1.17) | 0.30 |

CHR: cause-specific hazard ratio; CI: confidence interval

*For prior treatment: fully adjusted CHR is stratified on study site and tumour stage (early/late), and adjusted on age (continuous), BMI (categorical), and education (continuous)

†For Prior surgery, prior radiotherapy, prior chemotherapy and prior endocrine therapy: fully adjusted CHRs are stratified on study site and tumour stage (early/late), and adjusted on age (continuous), BMI (categorical) education (continuous), and mutually adjusted on each specific treatment type
